# How to treat major depressive disorder with shorter-duration hypomanic episodes? A case report

**DOI:** 10.3389/fpsyt.2024.1411882

**Published:** 2024-07-15

**Authors:** Jiashu Yao, Shenpang Wang, Yifei Li, Jiating Xu, Ruihuan Ye, Yuedi Shen, Wei Chen, Ning Dai

**Affiliations:** ^1^ Department of Psychiatry, Sir Run Run Shaw Hospital, School of Medicine, Zhejiang University, Hangzhou, Zhejiang, China; ^2^ Department of Psychiatry, Shaoxing 7th People’s Hospital, Shaoxing, Zhejiang, China; ^3^ The Second Department of General Psychiatry, The Third Hospital of Quzhou City, Quzhou, Zhejiang, China; ^4^ The Affiliated Hospital of Hangzhou Normal University, Hangzhou Normal University, Hangzhou, Zhejiang, China; ^5^ Department of Gastroenterology, Sir Run Run Shaw Hospital, School of Medicine, Zhejiang University, Hangzhou, Zhejiang, China

**Keywords:** major depressive disorder with shorter-duration hypomanic episodes, bipolar spectrum disorder, subthreshold bipolar disorder, COVID-19, treatment

## Abstract

Here we report on a case of a 61-year-old female patient with 7-year history of major depressive disorder with shorter-duration hypomanic episodes who was prescribed with antidepressants which turned out to be ineffective. After a COVID-19 infection, the patient’s clinical presentation became sufficient for the diagnosis of bipolar disorder and she was consistently effective on a mood stabilizer and an atypical antipsychotic. The course of treatment in this case suggests bipolar disorder is not a binary disorder, but a continuous spectrum disorder. For patients suffering from major depressive disorder with shorter-duration hypomanic episodes, mood stabilizers and atypical antipsychotics are possibly more suitable than antidepressants.

## Introduction

It has been estimated that 30–55% of patients of major depressive disorder (MDD) have subthreshold hypomania (1–5). With increasing evidences, scholars believe that bipolar disorder is not a binary disorder, but a continuous spectrum disorder (6–13). Therefore, the DSM-5 adds other specified bipolar disorder (including short duration hypomanic syndromes and major depressive episodes, hypomanic symptoms and major depressive episodes) to the traditional subtypes of bipolar I disorder and bipolar II disorder (14). The DSM-5-TR continues to retain this categorization (15).

Although there have been some updates in the clinical categorization of bipolar disorder, very few guidelines recommend treatment to subthreshold bipolar disorder due to the lack of study. Therefore, it is difficult for psychiatrists to prescribe medications to these patients. The following case may have some implications for the treatment of patients with subthreshold bipolar disorder.

## History of present illness

A 61-year-old female was admitted to our emergency room due to overdose. After the death of a loved one 7 years ago, the patient experienced low mood, frequent crying, anhedonia, lack of energy and strength, back and neck pain, decreased appetite, feeling that life was meaningless, but denial of suicidal thoughts, plans, and behaviors. At that time, she was hospitalized in a psychiatric hospital, diagnosed with MDD, and was given duloxetine 120mg once a day, clonazepam 2mg once before bed. After adequate treatment, her depressive symptoms (e.g., low mood and anhedonia) were alleviated.

However, despite regular medication after discharge from the hospital, duloxetine became less and less effective. Over the past 7 years, the patient’s mood was unstable, being quite happy before and then becoming depressed. Sometimes she became very happy and talkative, willing to participate in social activities, but also got irritable and keen to spend money and drink alcohol. The husband was able to recognize that the patient’s elevated state was beyond normal, but it usually lasted for a few hours, at the most for a day. Over the past 7 years, psychiatrists had prescribed her antidepressants other than duloxetine. She could not recall the names of these antidepressants because they were not effective and were used for short period of time. 5 months ago, after a COVID-19 infection, there is a sudden change to the patient’s symptoms. Her family described that she changed to a different person, became excessively high, talkative, overenergetic even after only 4-hour sleep a day, and also very confident but verbally aggressive and easy to lose her temper. She started to participate in all kinds of activities and spent a lot of money on purchasing things and couldn’t stay at home. This situation lasted for 2 months and the patient stopped taking medication because she felt cured and did not need it anymore. 2 months ago, she became depressed again, lost interest in life, felt weak and started to having suicidal thoughts. Symptoms did not improve after taking duloxetine 120mg once a day. 2 days ago, the patient took more than 20 tablets of clonazepam to commit suicide, and was sent to the emergency room of our hospital, and then was sent to our department for inpatient treatment.

## Medical history

The patient underwent right ovariectomy for teratoma 30 years ago. She has a history of hyperlipidemia for more than 6 years, and has been taking atorvastatin 20mg qn for a long time, and is allergic to pollen and dust mites.

## Personal history

The patient is currently retired and was a prison staff member prior to retirement. She is introverted, sensitive, paranoid, has average interpersonal relationship, pursues perfection in her work, and keeps her home very clean and puts things in a specific place. Her family history revealed that her grandmother committed suicide by hanging, and her uncle has a history of mental disorder, details not available. She smoked (20 cigarettes/day) for decades and drank socially.

## Treatment and follow-up in the hospital

On admission, the patient’s cranial MR revealed scattered small ischemic foci in the frontal parietal lobes bilaterally. The score of 17 items of Hamilton Rating Scale for Depression (HAMD-17) was 22, and Hamilton Anxiety Rating Scale (HAMA) score was 23, Pittsburgh Sleep Quality Index (PSQI) score was 10.

The diagnosis was changed to bipolar disorder with a current major depressive episode. We chose the combination of lithium carbonate and quetiapine because the patient was at high risk for suicide and had poor sleep. Lithium carbonate was gradually increased to 0.3g three times a day (blood lithium concentration of 0.65 mmol/L was measured after 1 week), quetiapine was gradually increased to 0.3g once a night, and clonazepam 2 mg once before bed. Patient’s mood gradually improved after 9 days of hospitalization, and was reexamined for HAMD-17 (10 points) , HAMA (10 points) before discharge.

The patient was followed up for 6 months after discharge and was emotionally stable, with HAMD-17 and HAMA scores of 9, 8 (2 months after discharge), 7, 6 (4 months after discharge), and 8, 6 (6 months after discharge), respectively.

## Discussion

During the 7 years prior to infection with COVID-19, the patient’s diagnosis had been major depressive disorder, because the previous maximum 1-day period of hypomanic syndromes did not meet the diagnostic criteria for a hypomanic episode. Therefore, the treatment given by the psychiatrist was the antidepressant, which was apparently ineffective. After being infected by COVID-19, the patient’s manic symptoms became more severe and lasted for 2 months. After taking a mood stabilizer and an atypical antipsychotics, the patient’s depressive mood improved significantly and remained stable during the 6-month follow-up. Thus, from the above history characteristics, the patient should essentially have had bipolar disorder, not MDD. Two case series reported on a number of patients who had their first manic episode after infection with COVID-19, some of whom had a history of depressive episodes while others did not (16, 17).

Accumulating clinical phenomenological findings suggest that patients with major depressive episodes and shorter-duration hypomanic episodes represent a complex clinical phenotype that is perhaps best conceptualized as a continuum between patients with unipolar depressive episodes and those with bipolar II disorder as defined by DSM-5 (6–13). Angst J and colleagues (18) found that the clinical presentations of MDD patients with the subthreshold manic syndrome were more similar to those with bipolar II disorder than to MDD alone, whereas patients with only manic symptoms were intermediate between those with subthreshold manic syndrome and those with MDD alone. Parker G and colleagues (19) found that two groups of bipolar disorder and MDD with shorter-duration hypomanic episodes did not differ on age of onset of depressive and of hypomanic episodes, or by rates of depressive and bipolar conditions in first-degree family members. Moreover, two of their other studies illustrated that MDD patients with hypomanic episodes as short as 1 day were significantly different from patients with unipolar depression patients in terms of factors such as family history of hypomania, age of onset, mixed state and mood instability (8, 9). In a longitudinal observational study (20), 550 MDD patients with subthreshold hypomania were prospectively followed for a mean of 17.5 years. It was found that 19.6% of the sample experienced hypomania or mania, resulting in re-diagnosis to bipolar II disorder in 12.2% of patients and to bipolar I disorder in 7.5%. In three prospective studies of adolescents, varying proportions of MDD patients with shorter-duration hypomanic episodes had been converted to bipolar disorder (1, 21, 22). In addition, the patient in the case was a high-recurrence MDD patient prior to the diagnosis of bipolar disorder. A study has shown that high-recurrence and high-frequency MDD also predicts a high likelihood of bipolar spectrum disorder (23). In addition to the above clinical phenomenological studies, several biological studies of inflammatory cytokines, functional and diffusion magnetic resonance imaging proves that MDD with shorter-duration hypomanic episodes is a different phenotype from MDD (24–27).

To our knowledge, although there are no randomized controlled trial (RCT) studies of MDD and shorter-duration hypomanic episodes, there have been RCT studies of MDD with mixed features similar to the former. In a 6-week of double-blind treatment with either lurasidone at 20–60 mg/day or placebo, lurasidone was found effectively involved in patients with mixed features of MDD (28). For patients with mixed features of MDD, two guidelines recommend second-generation antipsychotics and mood stabilizers rather than antidepressants (29, 30).

This case may give us some hints that mood stabilizers and atypical antipsychotics may be more suitable than antidepressants for patients with major depressive disorder who have shorter-duration hypomanic episodes. Of course, more high-quality studies are needed in the future to confirm this conclusion.

## Data availability statement

The original contributions presented in the study are included in the article/Supplementary Material. Further inquiries can be directed to the corresponding authors.

## Ethics statement

Written informed consent was obtained from the individual(s) for the publication of any potentially identifiable images or data included in this article.

## Author contributions

JY: Writing – original draft. SW: Writing – original draft. YL: Writing – original draft. JX: Writing – original draft. RY: Data curation, Investigation, Writing – original draft. YS: Writing – review & editing. WC: Writing – review & editing. ND: Writing – review & editing.
